# Content Size-Dependent Alginate Microcapsule Formation Using Centrifugation to Eliminate Empty Microcapsules for On-Chip Imaging Cell Sorter Application

**DOI:** 10.3390/mi14010072

**Published:** 2022-12-27

**Authors:** Toshinosuke Akimoto, Kenji Yasuda

**Affiliations:** 1 Department of Pure and Applied Physics, Graduate School of Advanced Science and Engineering, Waseda University, 3-4-1 Okubo, Shinjuku, Tokyo 169-8555, Japan; 2 Department of Physics, School of Advanced Science and Engineering, Waseda University, 3-4-1 Okubo, Shinjuku, Tokyo 169-8555, Japan

**Keywords:** alginate microcapsule, size separation, single cell, surface charge density, zeta potential, permeability, reflective index, on-chip imaging cell sorter application

## Abstract

Alginate microcapsules are one of the attractive non-invasive platforms for handling individual cells and clusters, maintaining their isolation for further applications such as imaging cell sorter and single capsule qPCR. However, the conventional cell encapsulation techniques provide huge numbers of unnecessary empty homogeneous alginate microcapsules, which spend an excessive majority of the machine time on observations and analysis. Here, we developed a simple alginate cell encapsulation method to form content size-dependent alginate microcapsules to eliminate empty microcapsules using microcapillary centrifugation and filtration. Using this method, the formed calcium alginate microcapsules containing the HeLa cells were larger than 20m, and the other empty microcapsules were less than 3m under 4000 rpm centrifugation condition. We collected cell-containing alginate microcapsules by eliminating empty microcapsules from the microcapsule mixture with simple one-step filtration of a 20 m cell strainer. The electrical surface charge density and optical permeability of those cell-encapsulated alginate microcapsules were also evaluated. We found that the surface charge density of cell-encapsulated alginate microbeads is more than double that of cells, indicating that less voltage is required for electrical cell handling with thin alginate gel encapsulation of samples. The permeability of the alginate microcapsule was not improved by changing the reflective index of the medium buffer, such as adding alginate ester. However, the minimized thickness of the alginate gel envelope surrounding cells in the microcapsules did not degrade the detailed shapes of encapsulated cells. Those results confirmed the advantage of alginate encapsulation of cells with the centrifugation method as one of the desirable tools for imaging cell sorting applications.

## 1. Introduction

Cell separation and isolation are fundamental for single-cell-based analysis and further applications, such as using those single cells as parts of regenerative medicine. Because of its many advantages against conventional in vitro dish experiments, microdroplets are one of the suitable methods for isolating single cells for their analysis in minimal volumes. For example, microdroplets have an isolated compartment for each target cell identification and easy handling, prevention from spatial dispersion for quantitative analysis and further operation and a small reaction volume for faster concentration increase with minimum amounts of reagents [1]. The compartmentalization of single cells was accomplished using water microdroplets in mineral oil for biological analysis and was improved continuously for their more strict measurements [2,3,4,5]. This trend of microfluidics treatment for microdroplet handling and analysis expanded into digital microfluidics applications with the advent of the quantification of every single cell [6,7,8,9,10].

Following those water droplets, encapsulation technologies have also been developed to improve single-cell analysis. For example, microfluidics channels provide homogeneous polymers and gel capsules regardless of the difference of contents in those capsules [11,12,13,14,15,16,17,18,19,20,21,22,23]. In particular, gel microcapsules composed of biological polymers have many advantages against the microdroplets, such as temperature-dependent hydrogel [24] and alginate, where gelation reaction proceeds with divalent cations such as Ca^2+^ for cross-linking sodium alginate [25,26]. Several reports showed that alginate gel microcapsules could provide a suitable environment for encapsulated cells [27,28] and also for the single-cell polymerase chain reaction (PCR) platform as monoclonal, in analogy to a single-cellular clone [29,30]. One of the advantages of alginate gel microdroplets is their stability and capability to encapsulate. For example, alginate gel microdroplet PCR is a miniature scale of biological operation with fast heat transfer in a contamination-free environment with microdroplet structural stability. It is even capable of capturing the PCR products within the microdroplets. Moreover, another advantage of alginate gel microdroplets is their simple gel–sol exchange by chelating divalent cations with reagents such as ethylene-diamine-tetraacetic acid (EDTA), which enables us to capture/release target cells only by changing the divalent cations. Furthermore, exploiting the difference of affinity between EDTA and ethylene glycol tetraacetic acid (EGTA) to divalent cations, Ca^2+^-alginate gel microdroplets and Ba^2+^-alginate gel microdroplets were digested according to the difference in chelating efficiency of linkage-divalent cations of those chelating buffers [31]. We also adopted the quick gel–sol exchange of two-dimensional alginate sheets for the selective collection of target cells among candidate cultivated cells in agarose microchambers on alginate–collagen mixture substrate on a chip [32].

In this study, we aim to optimize an alginate encapsulation technology for imaging cell sorter applications because the cell encapsulation into alginate microcapsules can potentially maintain cells in isolated conditions even under the fully packed dense concentration and can prevent contamination during sorting procedures. In the conventional microfluidic alginate microcapsule formation methods, homogeneous same-size alginate capsules were formed regardless of the cells inside. Therefore, if we apply this encapsulation technology for screening, the total number of microparticles increases significantly more than the total number of cells. Hence, we developed a simple centrifugation method to acquire the alginate microcapsules containing cells with a cell strainer before the cell sorting procedure. With this method, the size of alginate microcapsules relies on the contents; the alginate microcapsules containing cells are larger than 10 μm whereas the vacant alginate microcapsules are 2 μm. We also have examined two significant concerns of alginate microcapsules for imaging cell sorter application; (1) whether surface changes of microcapsules give the stable electrophoretic force regardless of the condition of cells and (2) whether the visibility of encapsulated cells can be improved with modification of the refractive index of the surrounding medium. The results showed the alginate envelopes enhanced the surface charges of cells. However, the permeability of alginate capsules was not improved with the modification of the medium.

## 2. Principle

**Figure 1:**
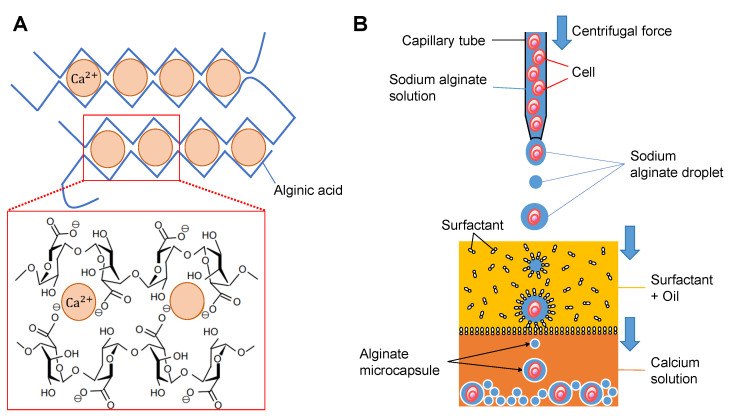
**Principle of alginate microcapsule formation.** (**A**). Schematic drawing of the change of soluble sodium alginate into insoluble calcium alginate. (**B**). Conceptual process of calcium alginate microcapsule formation. First, droplets of sodium alginate with/without cells are formed at the microcapillary tip with the pressure of centrifugal force. Then the droplets are introduced into the oil and coated with surfactants. Finally, the surfactant-coated droplets proceed to the calcium chloride solution and the surface of the sodium alginate droplet changes to insoluble calcium alginate as the alginate microcapsule.

Alginic acid is, in nature, a hydrophilic polysaccharide and forms soluble hydrogels with monovalent metal ions such as sodium and alginates. Alginates change to water-insoluble gelatinous calcium alginate by adding the aqueous calcium salt as described in Figure 1A and the following chemical reaction formula:

(1) $$2C_{6}H_{7}O_{6}\mathrm{Na}\;+\;\mathrm{Ca}{\mathrm{Cl}}_{2}\;\to \;{\left(C_{6}H_{7}O_{6}\right)}_{2}\mathrm{Ca}\;+\;2\;\mathrm{NaCl}.$$

Applying this soluble to insoluble gelation change of alginate droplet surfaces, we can acquire alginate microcapsules having an insoluble alginate layer surrounding the contents inside as solutions. For example, as shown in Figure 1B, after wrapping the surfactant monolayers on the surface of alginate droplets in the oil, the droplet is introduced into the calcium buffer. Then the surface of the droplets changed to insoluble gels surrounding the contents in the droplets. When we mix the cells or those clusters into the droplet buffer, single cells or single clusters are encapsulated into the microcapsules as far as the concentration of those cells and droplet size are appropriate.

## 3. Materials and Methods

### 3.1. Sodium Alginate Buffer

A mixture of 1% sodium alginate (80–120 cP; 194-13321, Wako, Osaka, Japan) and 5% glucose (Wako, Osaka, Japan) was prepared with mild stirring. The mixture was used as the sodium alginate buffer for alginate microcapsule formation.

### 3.2. HeLa Cells

In this experiment, we used HeLa cells (CCL-2™, ATCC, Manassas, VA, USA) as standard cells [33,34,35]. After we thawed the cryopreserved Hela cells, the cells were grown to 80% confluence in a 60 mm tissue culture dish (Techno Plastic Products AG, Trasadingen, Switzerland) at 37 ${}^{\circ }C$ under 5% CO_2_ in DMEM medium (Dulbecco’s modified Eagle’s medium, DMEM: Gibco, Thermo Fisher Scientific, Waltham, MA, USA) supplemented with 10% heat-inactivated fetal bovine serum (FBS: Gibco, Thermo Fisher Scientific, Waltham, MA, USA) and 100 U /mL penicillin-streptomycin (Gibco, Thermo Fisher Scientific, Waltham, MA, USA). After 4 min incubation with 0.25% trypsin- 1 mMEDTA (Thermo Fisher Scientific), we added 3~ 4 mL of DMEM medium and acquired sample cells from the supernatant passed through a 40 μm cell strainer (FALCON, Life Sciences, Durham, NC, USA). We washed the cells with the sodium alginate buffer in centrifugation three times and diluted them to the desired cell concentration for the alginate microcapsule experiments.

### 3.3. Microbeads

We used 10 μm YG fluorescent polystyrene microbeads (Fluoresbrite Carboxylate YG Microspheres, Polyscience, Inc., Warrington, PA, USA) as standard particle models. The beads were washed and exchanged into the sodium alginate buffer with mild centrifugation three times before using them to prevent aggregation.

### 3.4. Alginate Capsule Formation

First, we prepared a tapered glass micropipette having a 100 μm tip diameter for sodium alginate microdroplet formation. We loaded a capillary glass tube (GC150T-10, Harvard Apparatus, Cambridge, UK) into a micropipette puller (P-97/IVF, Sutter Instrument, Novato, CA, USA) and fabricated the micropipette having the desired hole diameter.

Next, the micropipette for sodium alginate droplet formation was prepared. Using a syringe (21G, Terumo Corporation, Tokyo, Japan), we filled glycerin (Wako, Osaka, Japan) from the tip to 0.36 cm of the glass micropipette for capping the end of the micropipette with this high viscous solution. Then, we decanted the sodium alginate buffer into the micropipette on the glycerin layer.

We also prepared the two-layer buffer reservoir, in which 50 μL of the calcium solution having 10% calcium chloride (Wako, Osaka, Japan) in 5% glucose solution was placed in a 1.5 mL sample tube (Eppendorf AG, Hamburg, Germany) as the bottom layer and 200 μL of the soybean oil (190-03776, Wako, Osaka, Japan) containing one percent surfactant (SY-Glyster CRS-75, 81003, SAKAMOTO, Osaka, Japan) was placed on the calcium solution layer as the top layer.

**Figure 2:**
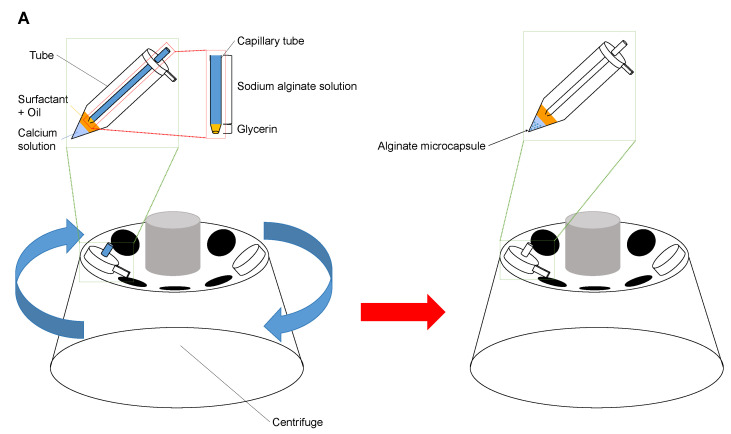

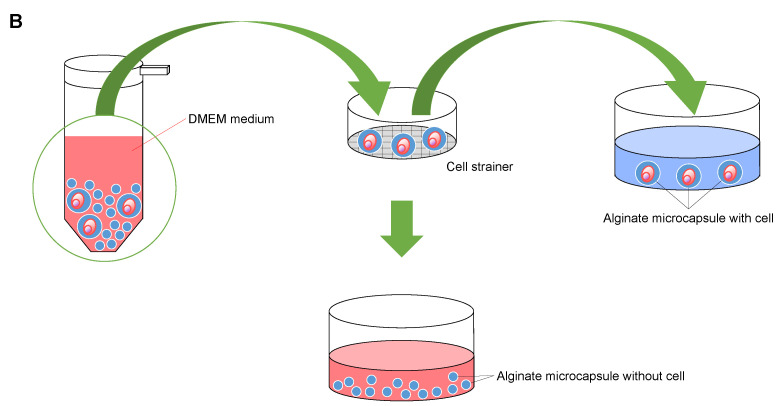
**Centrifuge method for production of calcium alginate microcapsules.** (**A**). Alginate microcapsule formation procedure. First, high viscous glycerin was filled into the tip of the tapered micropipette, then followed by the sodium alginate buffer containing cells. The micropipette was placed in the 1.5 mL tube. After capsules of sodium alginate solution are produced by centrifugal force, they change into alginate microcapsules. (**B**). Separation of alginate microcapsules containing cells using a cell strainer. The mixture of alginate microcapsules with/without cells was separated by using 20 μm cell strainer.

After setting the tip of the micropipette filled with sodium alginate on the two-layer buffer reservoir, we formed calcium alginate microcapsules as follows: First, as shown in Figure 2A, we set and fixed the glass micropipette at the lid of the 1.5 mL tube, where the tip of the micropipette attached to the upper soybean oil layer in the sample tube. Then we placed this combined micropipette and microtube setup in a centrifuge (Centrifuge 5424R, Eppendorf AG, Hamburg, Germany) and rotated to form alginate microdroplets at the tip of the micropipette. Finally, we acquired alginate microcapsules in the bottom calcium solution layer in the microtube. We added microbeads or Hela cells into the sodium alginate buffer to acquire calcium alginate microcapsules containing microbeads or HeLa cells.

Next, as shown in Figure 2B, we transferred alginate capsules into a DMEM medium and filtrated them through the 20 μm cell strainer (plurStrainer Mini 20 μm, plurStrainer Life Science, Leipzig, Germany). Then, we acquired alginate microcapsules containing cells only as supernatant of the cell strainer.

The images of samples were observed with the inverted microscope (IX71, Olympus, Tokyo, Japan) and recorded with a CCD camera (1501M-GE, CCD camera; Thorlabs, Newton, NJ, USA). We measured the particle size from the acquired images by using the image analysis software ImageJ (NIH, Bethesda, MD, USA) and then calculated the frequency distribution.

### 3.5. Surface Charge Measurement of Particles

Two carbon rod electrodes (ϕ 0.5 cm, Kenis, Osaka, Japan) were set in parallel with a distance of 2.4 cm in the 35 mm dish (AGC TECHNO GLASS Co., Ltd., Shizuoka, Japan). Two wires were connected to the AC voltage source (Function/Arbitrary Waveform Generator 33120A, Keysight Technologies, Santarosa, CA, USA and High-Speed Bipolar Amplifier BA4825, NF Co., Kanagawa, Japan). We filled 2 mL of 5% glucose buffer into the dish and then set samples into the buffer. After applying AC electric field (0.5 Hz, 0.42~12.5 v/cm) to the electrodes, we measured the distance of oscillation of particles on the stage of the inverted microscope (IX70, Olympus, Tokyo, Japan) and recorded with a CCD camera (1501M-GE-TE, CCD camera; Thorlabs, Newton, NJ, USA). The distance of oscillation was measured with the image analysis software ImageJ (NIH, Bethesda, MD, USA).

### 3.6. Statistical Analysis

All statistical values of particle sizes and their movements were acquired as mean ± standard deviation (S.D.) of 100 particle samples for each experiment. The result of fitting linear equations to a set of data points was achieved using ordinary least squares regression (OLS) in Microsoft Excel (Microsoft Co., Redmond, WA, USA).

## 4. Results and Discussion

### 4.1. Centrifugation Method for Content-Size Dependent Alginate Microcapsule Formation

In the popular microfluidic pathway-based alginate encapsulation technology, the homogeneous same-sized alginate microcapsules are formed for a stable reagent reaction environment regardless of the cells inside. However, this approach forms many empty alginate microdroplets during cell encapsulation in the microfluidic pathway. Moreover, because of the similarity of alginate microcapsule sizes, separating empty microcapsules and cell-encapsulated microcapsules from their shape difference is challenging. Therefore, to reduce the number of alginate microcapsules, some other method for eliminating empty microcapsules was required to reduce the number of microparticles screening.

To maximize those potential advantages of alginate encapsulation of cells for further analysis, such as imaging cell sorter applications, we developed a simple content size-dependent alginate microcapsule formation method to eliminate empty alginate microcapsules exploiting the surface tension and centrifugal force to determine the formed droplet sizes. The centrifugation method has been one of the popular methods to form alginate microcapsules before the microfluidic method becomes popular [36,37,38,39,40]. In that development, the centrifugation method aimed to form the homogeneous sized alginate microcapsules regardless of contents; this was the same as the aim of the microfluidic method. Hence, the contents’ dependence on alginate microcapsule size difference is caused by the difference of balance between the surface tension and centrifugal force generated by the contents in the droplets. Hence, we examined the method to enhance the size difference between empty alginate microcapsules and cell-encapsulated microcapsules to eliminate empty microcapsules.

Figure 1 and show the schematical drawing outline of the developed centrifugation method. As described above, in the sections titled Principle and Materials and Methods, we examined the structural optimization of alginate microcapsules exploiting centrifugal force and surface tension at the micrometer-sized microcapillary tip for droplet formation. In our centrifugation method, we filled a highly viscous solution, glycerin, at the end of the capillary glass tube to prevent undesired sodium alginate droplet formation before the rotation speed reached the desired velocity. Hence, the formation of sodium alginate droplets at the tip of the capillary tube was started under stable conditions after reaching the set speed.

**Figure 3:**
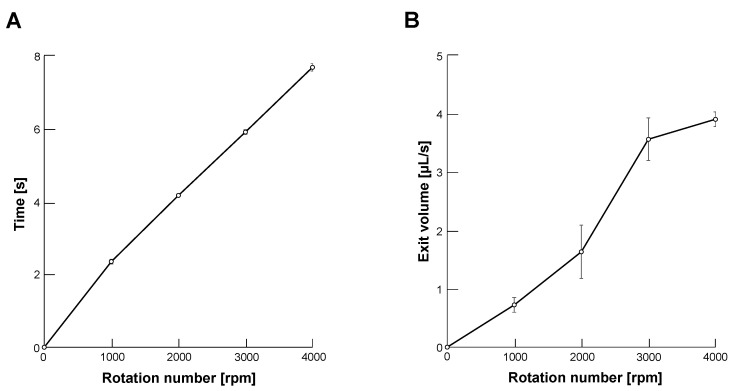
**Rotation number dependence of glycerin output from the tapered tip of microcapillary tube.** (**A**). The required time of centrifuge to set velocity after rotation started from 1000 to 4000 revolutions per minute (rpm) (n = 3). (**B**). Discharge volume of glycerin from the capillary tube in unit time (s) in each rotation number (n = 3).

First, we evaluated the output of glycerin from the end of the capillary tube with changing centrifugal force by controlling the rotation number of the centrifuge. Figure 3A shows the required time of the centrifuge to reach the desired velocities from 1000 to 4000 revolutions per minute (rpm) after rotation started. Figure 3B shows the discharge (exit) volume of glycerin in each rotation number. Applying these results, we determined the filled volume of glycerin at the tip region of the capillary tube. For example, we filled 32 μL glycerin at the tip of the capillary tube for 4000 rpm droplet formation. We determined the volume of glycerin in this condition 32 μL from a multiple of 8 s and 4 μL/s, which exceeds the volume of glycerin enough not to start sodium alginate microdroplet formation before the set velocity.

**Figure 4:**
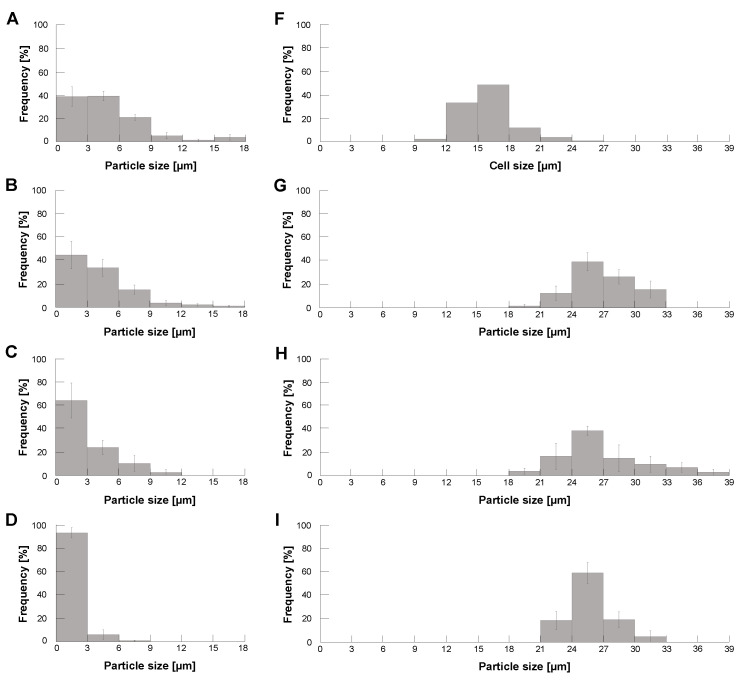

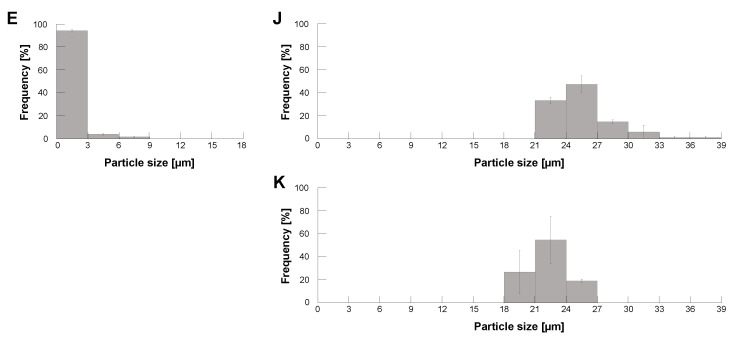
**Particle size distribution of calcium alginate microcapsules with/without HeLa cells under different centrifugal forces.** Size distribution of formed empty calcium alginate microcapsules (**A**–**E**), HeLa cells (**F**), formed calcium alginate microcapsules with HeLa cells (**G**–**J**), and formed calcium alginate microcapsules with 10μm polystyrene microbeads (**K**). (**A**). Formation of empty alginate microcapsules without glycerin capping for 1000 rpm. (**B**,**G**): 1000 rpm, (**C**,**H**): 2000 rpm, (**D**,**I**): 3000 rpm and (**E**,**J**,**K**): 4000 rpm (n = 3).

To confirm the contribution of glycerin caps to reduce the formation of unexpectedly large alginate microcapsules, we compared the size distribution of empty calcium alginate microcapsules with/without glycerin at the tip of the capillary tube. Figure 4A,B show the particle distribution of empty calcium alginate microcapsules with/without glycerin at the tip of the capillary tube. As expected, the graphs show that the ratio of larger microcapsules increased in Figure 4A. The results show that these larger alginate microcapsules were formed at a lower revolution speed before reaching the set revolution speed. Hence, the glycerin cap in the capillary tube successfully prevented the formation of those larger microcapsules.

Figure 4B–E show the revolution speed dependence of empty alginate microcapsule size distributions. As shown in the graphs, the size distribution of those empty microcapsules shifted to smaller as the revolution speed increased. Finally, they reached less than 10 μm both in 3000 rpm and 4000 rpm centrifugation.

Then, we examined the size distribution of HeLa cell-encapsulated alginate microparticles using the same centrifugation method. Figure F shows the size distribution of HeLa cells we used for encapsulation. Figure 4G–J also show the size distribution of HeLa cell-encapsulated alginate microparticles with different revolution speeds. As shown in those results, the size distribution of cell-encapsulated alginate microcapsules was independent of the revolution speed but mainly determined by those of encapsulated HeLa cells. Figure 4K shows the size distribution of 10 μm polystyrene microbead-encapsulated alginate microparticles in 4000 rpm. Comparing Figure 4J,K, the particle size of encapsulated samples was 10~ 12 μm larger than that of them, indicating the thickness of alginate gel envelope coated in this method is 5 μm both in HeLa capsules and polystyrene capsules.

The above results indicate that (1) the size of empty alginate microcapsules becomes smaller according to revolution velocity increase and (2) the size of HeLa cell-encapsulated alginate microcapsules maintains the identical size distributions reflecting the size distribution of HeLa cells regardless of the increase of revolution velocity. Hence, when we set the faster revolution velocity, the size distributions of empty alginate microcapsules and HeLa cell-encapsulated alginate microcapsules can be split into certain two-peak distributions with enough size gaps to separate them. For example, this experiment suggests that these two types of alginate microcapsules were divided easily into empty microcapsules less than 9 μm and cell-encapsulated microcapsules larger than 18 μm with the threshold size of 10 μm as far as we apply the revolution speed faster than 3000 rpm in the centrifugation method.

**Figure 5:**
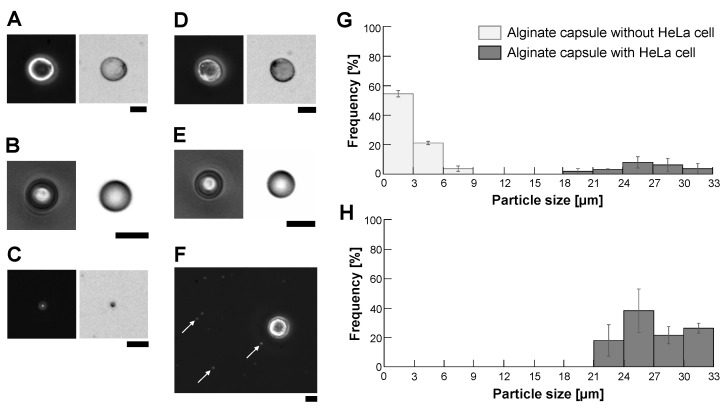
**Micrographs of empty and filled alginate microcapsules and their filtration results.** (**A**–**E**), Left: Phase-contrast image, Right: Bright-field image. (**A**): HeLa cell, (**B**): 10 μm fluorescent polystyrene microbead, (**C**): Empty alginate microcapsule, (**D**): HeLa cell-encapsulated alginate microcapsule, (**E**): 10 μm microbead-encapsulated alginate microcapsule. (**F**): Mixture of empty and HeLa cell-encapsulated alginate microcapsules acquired from sodium alginate buffer containing HeLa cells using a 4000 rpm centrifugation method. Arrows indicate the empty alginate microcapsules. Bars, 10 μm. (**G**): Size distribution of the mixture of empty and HeLa cell-filled alginate microcapsules using the 4000 rpm centrifugation method. Filled bars, the ratio of alginate microcapsules containing HeLa cells and open bars, the ratio of empty alginate microcapsules, respectively. (**H**): Size distribution of particles after filtration of alginate microcapsules with 20 μm cell strainer (n = 3).

Figure 5A–E show the phase-contrast and bright-field images of samples. We enclosed HeLa cells (Figure 5A) or 10 μm polystyrene microbeads (Figure 5B) into alginate microcapsules using centrifugation method under 4000 rpm condition. As shown in images of the empty alginate microcapsule (Figure 5C), the HeLa cell-encapsulated alginate microcapsule (Figure 5D) and the polystyrene microbead-encapsulated alginate microcapsule (Figure 5E), a thin alginate gel envelope was gathered to form the empty microcapsule or covered samples to form the filled microcapsules under the 4000 rpm centrifugation encapsulation procedure. Compared to the images of the HeLa cell microcapsule (Figure 5D) and the 10 μm polystyrene microbead microcapsule (Figure 5E), a thin alginate gel envelope wrapped those samples similarly regardless of the difference between cells and polymer particles.

Figure 5F shows one of the images of the formed empty microcapsules and HeLa cell-encapsulated microcapsules dispersed on the cultivation dish for counting their size distribution. As shown in the micrograph, we observed significant size differences between the empty and filled microcapsules. Their size distribution is summarized in Figure 5G. As shown in the graph, the size of empty microbeads was less than 9 μm (open bars) and the filled microbeads were almost larger than 21 μm. These two peak distributions were as predicted by the results in Figure 4. More than 79% of the particles were empty alginate microcapsules in a mixture of the empty microcapsules and the filled microcapsules under 4000 rpm conditions. Hence, eliminating those tiny empty alginate microcapsules is essential to improve the efficiency of imaging cell sorter analysis significantly because the time-consuming, unnecessary 79% of empty microcapsules were removed.

As the empty microcapsules distributed less than 9 μm and this distribution had enough size gap with respect to the distribution of HeLa cell-encapsulated microcapsules, which was almost larger than 21 μm, we removed empty alginate microcapsules from a mixture with a 20 μm cell strainer. Figure 5H shows the size distribution of particles on the filter of the cell strainer. The graph shows that we acquired HeLa cell-encapsulated microcapsules effectively with simple filtration.

**Figure 6:**
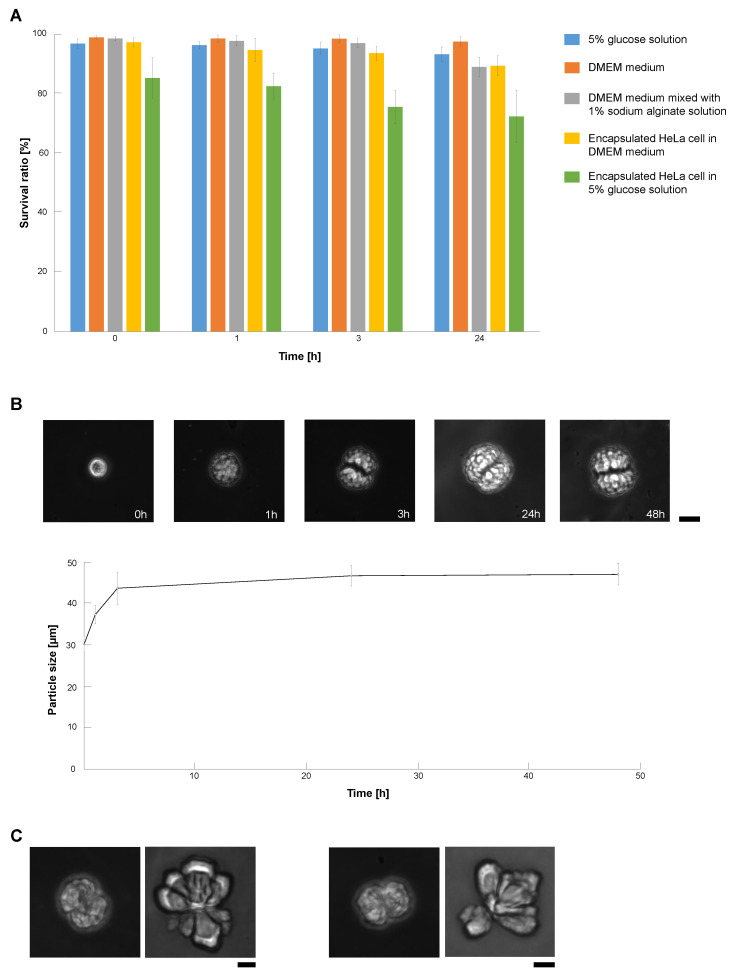
**Survival ratio and growth of HeLa cells in alginate microcapsules.** (**A**). Comparison of survival ratio of HeLa cells in DMEM medium and 5% glucose solution with (n = 3)/without (n = 5) alginate encapsulation. (**B**). Size increase of HeLa cell-encapsulated alginate microcapsules during cultivation (n = 3). Upper images are examples of cultivating microcapsules. Bar, 20 μm. (**C**). Encapsulated cells were released by exchanging calcium alginate gel for alginate sol in EDTA buffer. Left: Encapsulated HeLa cell in DMEM medium, Right: Encapsulated HeLa cell in EDTA buffer. Bar, 10 μm.

We evaluated the potential damage of HeLa cells in alginate microcapsules to estimate the allowed time for this sample microcapsule filtration and subsequent cell sorting procedure. We used two types of inside solutions of alginate microcapsules, DMEM medium and 5% glucose mixed with 1% sodium alginate solution. As described in Figure 6A, the encapsulation process in the centrifugation method caused little damage to HeLa cells in the microcapsules in the DMEM medium (Time = 0). The survival ratio of HeLa cells in the DMEM medium microcapsules maintained high values, similar to the dish cultivation samples. However, the survival ratio of HeLa cells in 5% glucose microcapsules decreased significantly according to the time passage and reduced to 70% after 24 h cultivation in those microcapsules. In contrast, as the survival ratio of cells in the same 5% glucose buffer in the cultivation dish remained higher than 93%, this loss of survival ratio was caused by the 5% glucose encapsulation. These results indicate that the use of DMEM medium for microcapsule experiments is recommended and if we use 5% glucose, quick and faster investigations are advised to maintain the higher survival ratio of cells.

Figure 6B also shows the time course change of HeLa cell-encapsulated alginate microcapsules. As shown in the graph and micrographs, the alginate microcapsules grew according to the growth and cell divisions of HeLa cells inside the microcapsules up to 1.5 times larger than their original sizes within 24 h and maintained their sizes even after 48 h cultivation.

We used EDTA to release encapsulated HeLa cells because EDTA can chelate divalent cations and exchange calcium alginate gel for alginate sol. We soaked encapsulated HeLa cells in 10 mM EDTA (2NA, Dojindo, Kumamoto, Japan) buffer (pH = 8) for 15 min. Figure 6C shows examples that HeLa cells in the alginate capsules (left images) were released from the capsules (right images) in the EDTA buffer.

### 4.2. Electric Charge of Alginate Capsules: Content Dependency

Another potential advantage of alginate encapsulation of cells is providing a stable surface change of alginate envelope to samples. We can use these stable surface charges of microcapsules for handling samples regardless of the difference in contents. Hence, we evaluated the values of surface charges of alginate microcapsules and their encapsulated particle dependences.

We estimated the effective electric charge density of the microparticles from the amplitude of the sample oscillations in the AC electric field as follows. When we place a spherical particle with a charge *q* and a radius *r*, the amplitude of oscillation *L* in the AC electric field Esinωt is described as,

(2) $$\begin{array}{ccc} m\frac{\partial^{2}x}{\partial t^{2}} & = & 0=qE\sin\omega t-6\pi \eta rv \end{array}$$

(3) $$\begin{array}{ccc} v & = & \frac{qE}{6\pi \eta r}\sin\omega t \end{array}$$

(4) $$\begin{array}{ccc} L\;=\;\int_{0}^{\frac{T}{2}}v\;dt & = & \frac{qE}{6\pi \eta r}\int_{0}^{\frac{T}{2}}\sin\omega t\;dt=\;\frac{q}{3\pi \eta r}E \end{array}$$

where *T* is the cycle period, which is slow enough to balance the motion and viscous resistance during the entire oscillation. Therefore, the surface charge density γ of the spherical particle is replotted as,

(5) $$\begin{array}{ccc} q\;=\;4\pi r^{2}\gamma & = & \frac{3\pi \eta L}{E}r \end{array}$$

(6) $$\begin{array}{ccc} ∴\;\gamma & = & \frac{3\eta }{4r}\frac{L}{E}. \end{array}$$

Hence, we used Equation (6) to estimate the surface charge density γ from the measured amplitude of oscillation *L* in the experiment.

**Figure 7:**
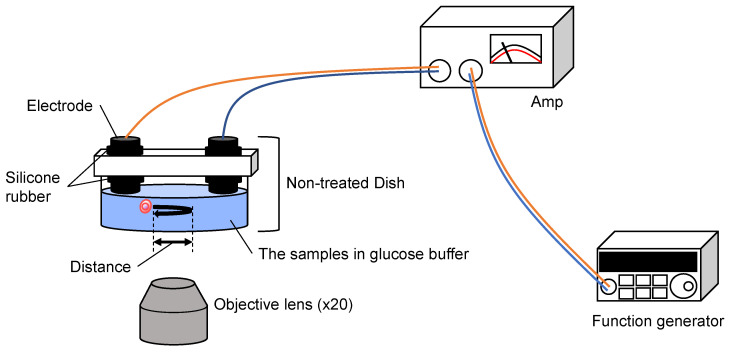
**Setup of the sample oscillation measurement system.** AC electric field (0.5 Hz, 0.42~12.5 v/cm) was applied between the two carbon rod electrodes from the function generator and the amplifier. The oscillation of samples was measured with optical microscopy.

We placed particles in 5% glucose solution and applied 12.5 vpp/cm, 0.5 Hz AC electric field in the sample oscillation measurement system as shown in Figure 7. In this experiment, we used 5% glucose solution as the medium instead of the cultivation buffer such as DMEM to maximize the electric force for handling cells, just the same as the cell sorting buffer in our imaging cell sorter system [41,42].

**Figure 8:**
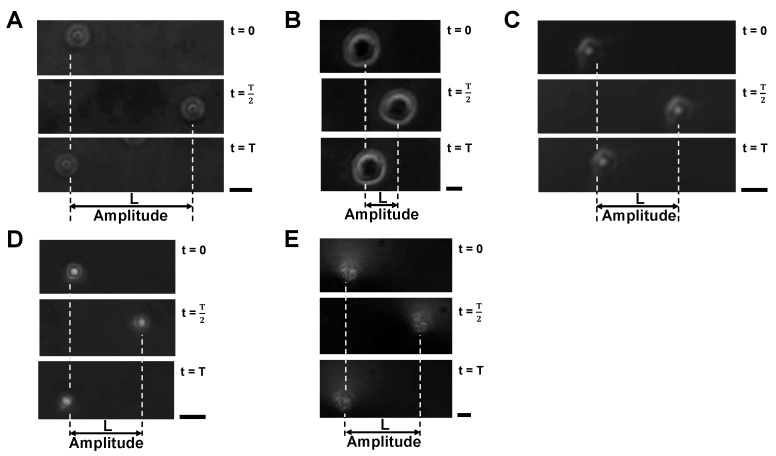
**Oscillation amplitudes of samples in 12.5 vpp/cm, 0.5 Hz AC electric field.** (**A**): 10 μm polystyrene microbead, (**B**): HeLa cell, (**C**): Empty alginate microcapsule, (**D**): 10 μm polystyrene microbead-encapsulated alginate microcapsule, (**E**): HeLa cell-encapsuled alginate microcapsule. All the experiments were performed in 5% glucose solution. Bars, 10 μm.

Figure 8 shows examples of oscillating samples; 10 μm polystyrene microbead (A), HeLa cell (B), empty alginate microcapsule (C), 10 μm polystyrene microbead-encapsulated alginate microcapsule (D) and HeLa cell-encapsuled alginate microcapsule (E). Upper, middle and lower micrographs show the positions of samples at t=0, t=T/2 and t=T, respectively. As shown in those micrographs, the particles moved from the original positions to the peak distance *L* at t=T/2 and then returned to the original position again at t=T. Hence, we used acquired oscillation amplitude distance *L* to estimate surface charge density γ.

**Figure 9:**
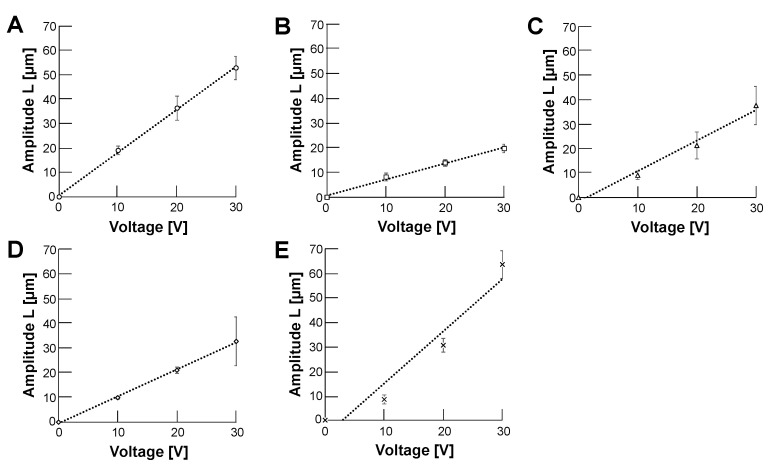
**The relationship between AC voltage and oscillation amplitudes of samples.** The applied frequency was 0.5 Hz in 5% glucose solution. (**A**): 10 μm polystyrene microbead, (**B**): HeLa cell, (**C**): Empty alginate microcapsule, (**D**): 10 μm polystyrene microbead-encapsulated alginate microcapsule (**E**): HeLa cell-encapsuled alginate microcapsule. Error bars, standard deviations (SDs). The result of fitting linear equations to a set of data points was achieved using ordinary least squares regression (OLS) in Microsoft Excel (Microsoft Co., Redmond, WA, USA) (n = 3).

Figure 9 shows the applied AC voltage intensity dependence of oscillation amplitude distance change in samples; 10 μm polystyrene microbead (A), HeLa cell (B), empty alginate microcapsule (C), 10 μm polystyrene microbead-encapsulated alginate microcapsule (D) and HeLa cell-encapsulated alginate microcapsule (E). The amplitudes of oscillation amplitude distances in all the samples were directly proportional to the amplitude of applied AC voltages, which satisfies Equation (4). Therefore, we can estimate the surface charge density γ from the slope of the fitting linear lines by applying Equation (6).

**Figure 10:**
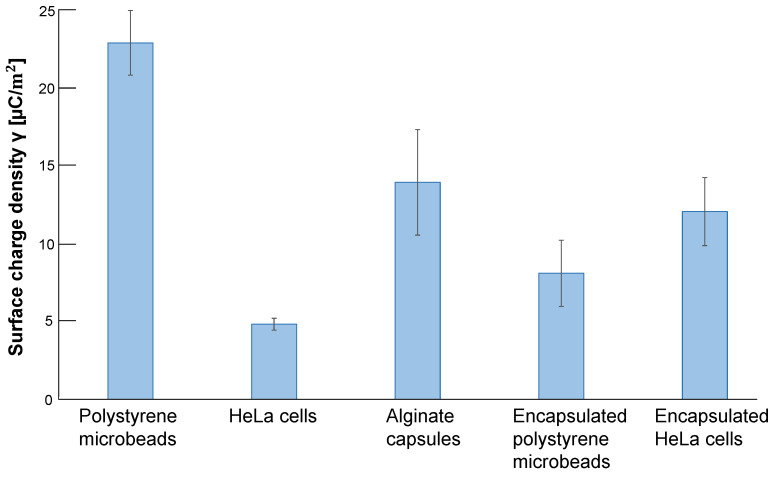
**Surface charge densities of samples estimated by the slope of AC voltage and oscillation amplitude relationship.** Surface charge density of 10μm polystyrene microbeads, HeLa cells, empty alginate microcapsules, 10 μm polystyrene microbead-encapsulated alginate microcapsules and HeLa cell-encapsulated alginate microcapsules. Error bars, SD (n = 3).

Figure 10 shows the summary of surface charge densities of samples acquired from the results in Figure 9. The graph shows that the surface charge density of polystyrene spheres was the largest in all five samples. However, contrary to expectations, the surface charge density of alginate gel-covered polystyrene spheres decreased by less than half, even smaller than those of the empty alginate microcapsules. In contrast, the surface charge density of HeLa cells was increased more than two times by alginate gel wrapping as microcapsules. It is even more significant than the charge density of polystyrene bead-encapsulated alginate microcapsules.

As described above, enhancement of the surface charge of cells using an alginate gel envelope was successful in HeLa cells. This is one of the potential advantages of alginate microcapsules for imaging cell sorter applications because we can reduce the voltage of handling samples in microfluidic pathways for separation. However, we need to mention the possibility of opposite results of surface charge density and its reduction in polystyrene spheres for practical applications. Although the ζ-potential of alginate beads was reported in many works [43,44], the influence of content particles on the ζ-potential of alginate microcapsule surfaces has not been investigated yet. We are also still uncertain about the origin of these paradoxical sample-dependent differences.

One possible reason for charge density reduction might be the interference of the thin alginate gel layer to reduce the thickness of the electric bilayer formed on the surface of polystyrene spheres. In contrast, the increase of the surface charge of HeLa cells by encapsulation also might be caused by the assistance of the surface charge density of alginate gel because both the surface charge and the thickness of the electric bilayer of HeLa cells were significantly smaller than the alginate gel. High values of empty alginate microbeads also might be caused by the significant size difference of empty alginate capsules from HeLa cells and polystyrene spheres. Equation (6) shows that the surface charge density estimation depends on the particles’ size. If the size contribution is more significant than expected, the estimated value of empty microcapsules might equal that of the HeLa cell-encapsulated alginate microcapsules.

### 4.3. Image Recognition of Single Cells Encapsulated in Alginate Beads

For the imaging cell sorter application of alginate microcapsules, there is improvement in the permeability of the thin alginate gel layer surrounding samples in the microcapsule envelopes with regard to more precise observation in an imaging cell sorter. Hence, we examined the improvement of permeability of alginate microcapsules and encapsulated HeLa cells by optimizing the refractive index of the buffer.

**Figure 11:**
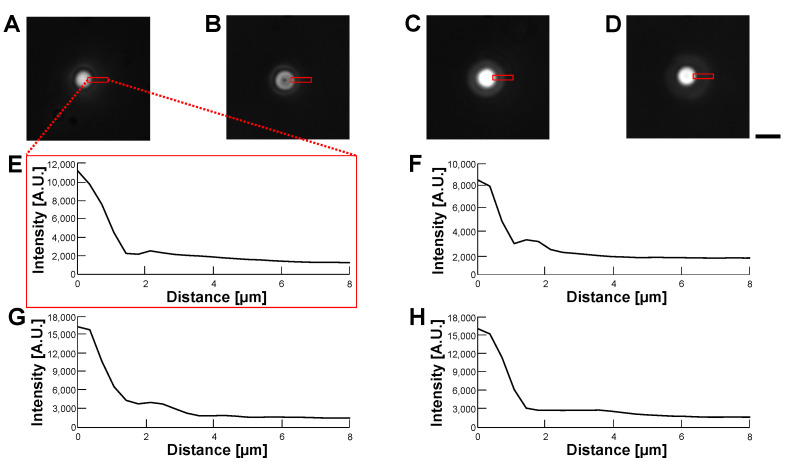
**Comparision of permeability of empty alginate microcapsules in various buffer conditions by phase-contrast observation.** (**A**–**D**): Phase-contrast images of empty alginate microcapsules. (**A**): 5% glucose solution (**B**): 10% glucose solution (**C**): 15% glucose solution (**D**): 1% alginate ester. (**E**–**H**): Intensity profiles of empty alginate microcapsule images (**A**–**D**), respectively. Bar, 10 μm.

**Figure 12:**
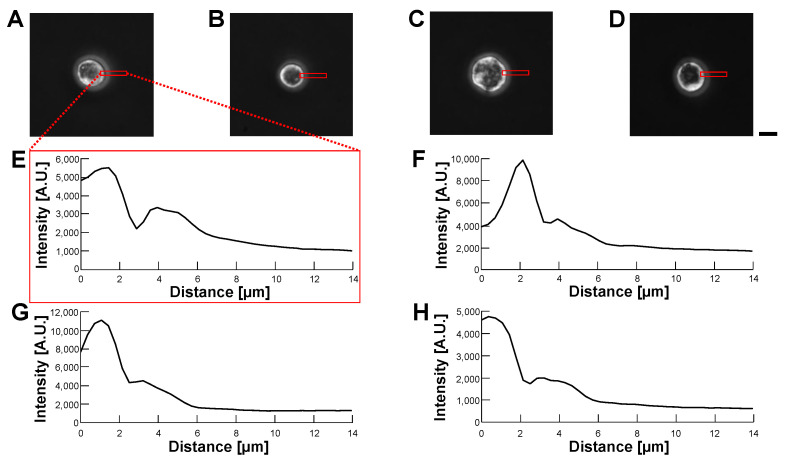
**Comparision of permeability of HeLa cell-encapsulated alginate microcapsules in various buffer conditions by phase-contrast observation.** (**A**–**D**): Phase-contrast images of HeLa cell-encapsulated alginate microcapsules. (**A**): 5% glucose solution (**B**): 10% glucose solution (**C**): 15% glucose solution (**D**): 1% alginate ester. (**E**–**H**): Intensity profiles of HeLa cell-encapsulated alginate microcapsule images (**A**–**D**), respectively. Bar, 10 μm.

Figure 11 and Figure 12 show the phase-contrast images of empty alginate microcapsules (Figure 11) and HeLa cell-encapsulated alginate microcapsules (Figure 12) in various buffer conditions: 5% glucose solution (A), 10% glucose solution (B), 15% glucose solution (C) and 1% alginate ester (D). As shown in the phase-contrast micrographs and their intensity profiles of empty alginate microcapsules (Figure 11), the border of microcapsules was not permeable in all buffers. We observed the edge of the particle even in the 1% alginate ester buffer, which is a similar component to the alginate gel microcapsule. This appearance of the edge image should be caused by the sensitivity of phase-contrast microscopy to the structural difference of macromolecule polymers. In contrast, the phase-contrast micrographs and their intensity profiles of HeLa cell-encapsulated microcapsules indicate the apparent borders of cells in the microcapsules and no significant influence on cell image acquisition was observed regardless of the buffers.

**Figure 13:**
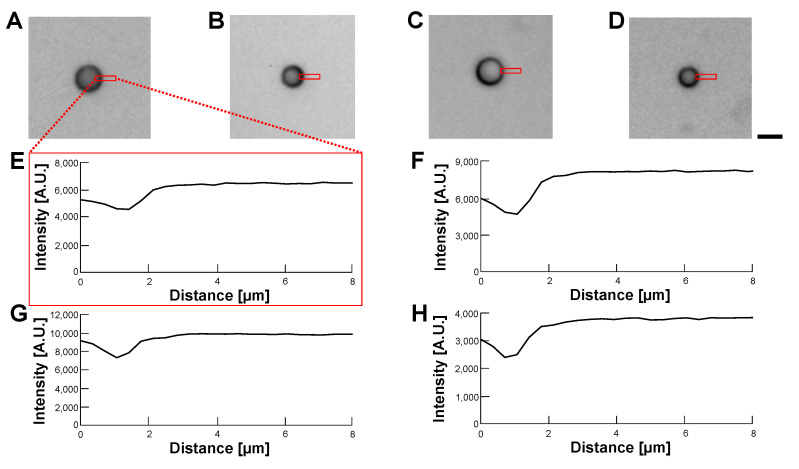
**Comparision of permeability of empty alginate microcapsules in various buffer conditions by bright-field observation.** (**A**–**D**): Bright-field images of empty alginate microcapsules. (**A**): 5% glucose solution (**B**): 10% glucose solution (**C**): 15% glucose solution (**D**): 1% alginate ester. (**E**–**H**): Intensity profiles of empty alginate microcapsule images (**A**–**D**), respectively. Bar, 10 μm.

**Figure 14:**
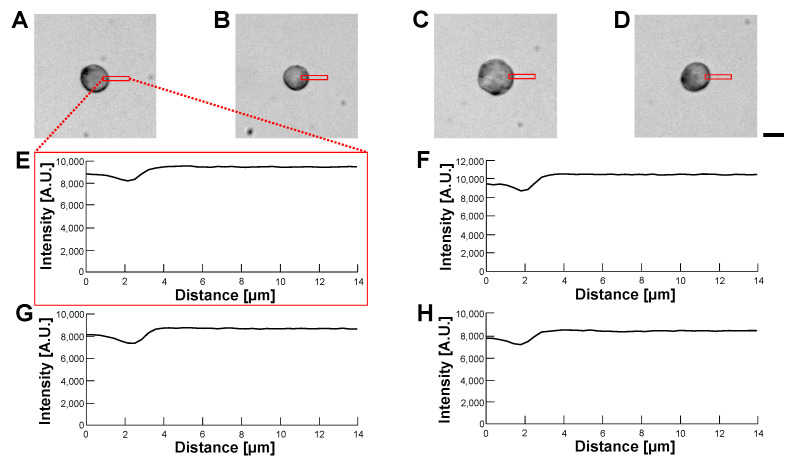
**Comparision of permeability of HeLa cell-encapsulated alginate microcapsules in various buffer conditions by bright-field observation.** (**A**–**D**): Bright-field images of HeLa cell-encapsulated alginate microcapsules. (**A**): 5% glucose solution (**B**): 10% glucose solution (**C**): 15% glucose solution (**D**): 1% alginate ester. (**E**–**H**): Intensity profiles of HeLa cell-encapsulated alginate microcapsules images (**A**–**D**), respectively. Bar, 10 μm.

Figure 13 and Figure 14 also show the bright-field images of empty alginate microcapsules (Figure 13) and HeLa cell-encapsulated alginate microcapsules (Figure 14) in various buffer conditions the same as in Figure 11 and Figure 12. As shown in these bright-field micrographs and their intensity profiles of empty alginate microcapsules (Figure 13), the border of microcapsules was observed as the difference of intensities in all buffers regardless of their differences even in the 1% alginate ester buffer. Hence, the buffers’ refractive index change did not successfully make transparent alginate microcapsules even in the bright-field observation. However, just like in phase-contrast images, the bright-field images and intensity profiles of HeLa cells in HeLa cell-encapsulated microcapsules show more significant clear images of cell edges and the alginate envelope did not appear even in the intensity profiles (Figure 14).

According to the above results, we concluded that the optimization of the refractive index of the buffer was not effective in making transparent alginate envelopes even in the 1% alginate ester buffer. However, the alginate envelopes surrounding HeLa cells did not degrade those cells’ images in phase-contrast and bright-field observations. This might be caused by the advantage of the centrifugation method, in which a minimum thin alginate gel envelope enclosed HeLa cells.

## 5. Conclusions

We investigated the ability of alginate gel microcapsules to image cell sorter applications. In this study, we examined the possibility of forming a homogeneous thin alginate gel envelope on the cells exploiting the balance of two opposite forces, centrifugation force and viscose force. For this experiment, we developed a simple droplet formation method as the centrifugation method exploiting the micrometer-sized tip of the capillary tubes in the centrifuge. The results showed that the homogeneous envelope was coated surrounding the cells and empty alginate microcapsules were less than a few micrometers. The centrifugation method accomplished the size-dependent selective collection of alginate microcapsules containing cells at 4000 rpm centrifugation and filtration. Significant enhancement of surface charge density of HeLa cells by the thin alginate gel encapsulation on the surface of cells was also observed. Change in the refractive index of the buffer did not improve the permeability of alginate envelopes; however, the advantage of the centrifugation method for wrapping minimum thickness of alginate gel surrounding cells can cause the clear phase-contrast and bright-field images of cells with a negligible small influence of alginate gels. All the above results suggest the alginate encapsulation of cells can give more advantages to an imaging cell sorter, such as (1) maximized concentration of samples maintaining their isolated conditions in microcapsules without any empty capsules, (2) enhancement of surface charge of cells for easy handling of electrostatic force and (3) improvement of permeability for observation of contents in the alginate microcapsules with minimized alginate gel envelope thickness.

## Data Availability

Not applicable.
